# *ABCA1* Polymorphism R1587K in Chronic Hepatitis C Is Gender-Specific and Modulates Liver Disease Severity through Its Influence on Cholesterol Metabolism and Liver Function: A Preliminary Study

**DOI:** 10.3390/genes13112095

**Published:** 2022-11-11

**Authors:** Joana Ferreira, Manuel Bicho, Fátima Serejo

**Affiliations:** 1 Institute for Scientific Research Bento Rocha Cabral, 1250-012 Lisbon, Portugal; 2 Environmental Health Institute (ISAMB), Genetics Laboratory, Lisbon Medical School, University of Lisbon, 1649-004 Lisbon, Portugal; 3 Gastroenterology and Hepatology Department, Hospital de Santa Maria, 1649-028 Lisbon, Portugal

**Keywords:** chronic hepatitis C, fibrosis, ATP binding cassette transporter A1, genetic polymorphism

## Abstract

Chronic hepatitis C (CHC) progression is highly variable and can be influenced by lipid metabolism. The ATP-binding cassette transporter A1 (ABCA1) is involved in lipid metabolism and mediates cholesterol efflux from liver cells. *ABCA1* gene polymorphism rs2230808 (R1587K) modulates lipid levels as it is located in an ABCA1 protein domain, which is essential for cholesterol efflux. We aimed to analyze the role of *ABCA1* polymorphism R1587K (rs2230808) in modulating the biochemical parameters of lipid metabolism and liver function and its association with liver disease severity, according to gender. A total of 161 CHC patients were clinically, histologically, and biochemically evaluated. Genotyping was performed by melting-curve analysis and statistical analysis by SPSS 24.0. There were significant differences between *ABCA1*_rs2230808 genotypes and total cholesterol, γGT (γ-glutamyl-transpeptidase), and HCV-RNA. Gender differences: in females, *ABCA1*_rs2230808 (GG or GA) was associated with higher HCV-RNA serum levels; in males, *ABCA1*_rs2230808 (GG or GA) was associated with higher γGT, lower total cholesterol, increased risk for γGT ≥ 38 UI/L, and total cholesterol < 4.92 mmol/L. Only in the case of males were higher γGT and lower total cholesterol associated with severe fibrosis and steatosis. Total cholesterol < 4.92 mmol/L also associates with severe necroinflammation. We conclude that *ABCA1*_rs2230808 is gender-specific. *ABCA1*_rs2230808 Allele G was associated with different clinical and biochemical parameters, which are related to more severe liver disease.

## 1. Introduction

If untreated, chronic hepatitis C (CHC) is a significant health and economic problem owing to life-threatening complications like portal hypertension, liver failure, or hepatocellular carcinoma [1]. In addition, epidemiologic data show that hepatitis C virus (HCV) infection accounts for liver-related morbidity and all-cause mortality twice that of HCV-negative infections [2].

Disease progression depends on factors such as the age of acquisition and duration of infection, gender, race, host genetics, viral factors, alcohol consumption, smoking, and metabolic factors [3]. It has been demonstrated that women have biochemical and histological evidence of less advanced liver disease [4]. This fact is related to estrogens’ anti-fibrotic properties and suggested by evidence that menopause is associated with fibrosis progression and that hormone replacement therapy may minimize this effect [5]. Identifying factors associated with slow versus rapid progression is a hot topic. 

The association between steatosis severity and stage of fibrosis is controversial [6,7]. In CHC, steatosis can be virally induced mainly in the HCV genotype three and metabolically generated in infections with other genotypes. Hepatic steatosis predominates in individuals infected with the HCV genotype [3], likely due to the specific effects of core proteins. It is also known that there is a direct correlation between hepatic steatosis and intrahepatic viral load in these individuals, suggesting an immediate impact of HCV that is not observed in the other HCV genotypes [8]. 

It is known that lipids have a pivotal role in the HCV life cycle. HCV is a lipid-containing virus that binds to low-density lipoprotein (LDL) and, to a minor degree, to high-density lipoprotein (HDL) [9]. Total cholesterol, triglycerides, HDL, and LDL were found to be decreased in HCV patients [10,11,12]. The HCV core protein and host genetic factors may play a role in these lipid abnormalities [13]. Genetic polymorphisms within proteins involved in lipid metabolism may explain the heterogeneity of various studies’ results [14,15].

ABCA1 (ATP-binding cassette transporter A1) is a protein involved in lipid metabolism. It mediates cholesterol and phospholipids efflux from cells to lipid-pool apolipoproteins [16]. Defects on the ABCA1 pathway have been associated with coronary artery disease and increased atherosclerotic lesions [17,18]. Its liver expression promotes the clearance of excess cholesterol and phospholipids from hepatocytes and reduces hepatic lipid accumulation [19,20].

The ATP-binding cassette transporter A1 is codified by the *ABCA1* gene located on the long arm of chromosome 9 at position 9q31.1.

Several gene polymorphisms were identified within the *ABCA1* gene, such as rs2230808 (R1587K), characterized by a G to A change in the DNA chain. That leads to substituting an Arginine (R) with a Lysine (K). It is located in the two primary extracellular loops of the ABCA1 protein, which are essential for interacting with apoA-I and cholesterol efflux15. However, studies that investigated the association of this polymorphism with the lipid profile have revealed conflicting results [21,22,23].

## 2. Objectives

We aimed to analyze the role of *ABCA1* polymorphism R1587K (rs2230808) in modulating biochemical parameters of lipid metabolism and liver function and its association with liver disease severity, according to gender.

## 3. Methods

We prospectively studied a group of 161 patients with CHC infection. The sample comprises 60 females (37.3%) and 101 males (62.7%) from an outpatient department, with a mean age of 46.14 ± 12.46 years. The overall baseline characteristics of the 161 patients studied are described in Table 1.

The subjects were selected, examined, adequately informed, and provided consent following the WMA Helsinki Declaration [24].

Inclusion criteria: positive RNA and anti-HCV antibodies for more than six months; absence of other chronic liver diseases based on liver enzymes (ALT, AST, and γGT) within reference values (see section Biochemical evaluation).

Biochemical evaluation: Serum biochemical parameters were evaluated using standard methods (reference values described): alkaline phosphatase (AP ≤ 129 UI/L), aspartate aminotransferase (AST ≤ 34 IU/L), alanine aminotransferase (ALT ≤ 49 IU/L), γ-glutamyl-transpeptidase (γGT < 38 IU/L), platelets count (≥150,000/µL), total cholesterol (≤4.92 mmol/L), HDL (≥1.04 mmol/L), LDL (≤2.8 mmol/L), and triglycerides, (≤1.69 mmol/L).

Serum HCV-RNA was evaluated by Real-Time PCR Taqman and genotypes by Hybridization Probes—LiPA “Line Probe Assay”.

DNA extraction: DNA was isolated from leukocytes by an adapted non-enzymatic DNA extraction procedure [25].

Genetic polymorphism identification: *ABCA1*_rs2230808 genotyping was performed by melting-curve analysis in a LightCycler 480II (Roche Diagnostics). The reaction included specific LightSnip assays (TIB MOLBIOL) and a LightCycler FastStart DNA Master Hybridization Probe (Roche Diagnostics). Each of the three possible genotypes result in different profiles of melting curves with different melting temperatures. *ABCA1*_rs2230808: Tm (G) = 64.00 °C, Tm (A) = 57.60 °C.

Histologic evaluation: A liver biopsy was performed on 99 patients using a Menghini needle [26]. Steatosis was assessed in 71 patients by the Brunt score [27] and staging/grading was assessed by the Metavir score [28] in 99 patients. Hepatic fibrosis was also evaluated by transient elastography (TE) in 101 patients using a FibroScan^®^ device (Echosens, Paris, France) with a 5 MHz ultrasound transducer mounted on the axis of a vibrator. The vibrator generates a painless vibration (frequency of 50 Hz and amplitude of 2 mm) like a “flick”, generating a shear wave that propagates through the skin and the subcutaneous tissue into the liver. The velocity of the wave is directly related to the LS. The median value of 10 successful acquisitions was expressed in kilopascals (kPa), with a success rate of at least 60% and an interquartile range (IQR) lower than 30%. Cut-off values were validated in the Gastroenterology and Hepatology Department, Hospital de Santa Maria, Lisbon, Portugal (analysis of 110 patients, Scheuer classification): 5.43 kPa for F ≥ 2 (PPV 0.78; NPV 0.67); 8.18 kPa for F ≥ 3 (PPV 0.95 NPV 0.93); 12.00 kPa for F = 4 (PPV 0.93; NPV 0.93) [29].

Statistical analysis:We used SPSS 24.0 to perform statistical analysis. Mean ± SD and median (min–max) were used to describe continuous standard and non-normal variables, respectively. Absolute and relative frequencies were applied for categorical variables. Bivariate analysis was performed by χ^2^ test and Odds Ratio for categorical variables, whereas for continuous variables we used independent samples *t*-Test, Mann-Whitney and ANOVA, or Kruskal-Wallis tests.

Differences were considered significant for *p*-values < 0.05.

## 4. Results

### 4.1. Baseline Clinical, Biochemical, and Histological Variables According to Gender

At enrollment, males’ ages were significantly lower, and γGT and HDL were significantly higher (Table 1).

Of all the serum biochemical parameters, more than 70% of individuals showed normal values.

*ABCA1*_rs2230808 genotype frequencies did not differ between genders.

### 4.2. ABCA1 Polymorphism According to All the Baseline Clinical, Biochemical, and Histological Variables

In males, we found significant differences in γGT (*p* = 0.033) and total cholesterol (*p* < 0.001) according to *ABCA1*_rs2230808 genotypes (Table 2). G allele carriers (GG or GA) had higher γGT (*p* = 0.017) and lower total cholesterol (*p* = 0.044) (Table 3). They also showed an increased risk for presenting γGT ≥ 38 UI/L (OR = 5.686 95% CI [1.338–24.159], *p* = 0.019) and total cholesterol ≤ 4.92 mmol/L (OR = 9.492 95% CI [1.679–53.656], *p* = 0.011). In females, G allele carriers (GG or GA) had a significantly higher HCV viral load as measured by total serum HCV-RNA *p* = 0.013 (Table 3).

### 4.3. ABCA1-Dependent Biochemical Variables and Correlation with the Severity of Liver Disease

Regarding biochemical variables associated with *ABCA1*_rs2230808 in univariate analysis, the HCV viral load in females was measured by total serum HCV-RNA and γGT and by total cholesterol in males (Table 2).

In females, we did not find significant differences (Table 4 (panel a)).

In males, significantly lower total cholesterol and higher γGT were found in patients with severe fibrosis (*p* = 0.024; *p* = 0.002) and steatosis (*p* = 0.019; *p* = 0.010) (Table 4 (panel b)). They also showed severe necroinflammation for total cholesterol ≤ 4.92 mmol/L (OR = 4.490, 95% CI [1.493–13.474], *p* = 0.007) and severe fibrosis for γGT ≥ 38 UI/L (OR = 4.572, 95% CI [1.685–12.406], *p* = 0.003).

## 5. Discussion

Our study showed that females and males differed in some clinical and biochemical parameters from the baseline, especially those associated with more severe disease. However, histological characteristics were similar in both genders.

Nevertheless, it is known that males have more severe forms of CHC. This fact depends on viral factors, host genetics, and metabolic backgrounds [3,30,31,32].

In comparison to females, we have found that males have significantly lower HDL levels, but no significant differences in fibrosis stage, necroinflammatory activity, and steatosis. Ramcharran D et al. found that low lipid serum levels in HCV correlated with steatosis, fibrosis progression, and nonresponse to treatment. Moreover, higher baseline LDL levels were good predictors of a sustained virologic response [33]. HCV infection was associated with an altered expression of genes involved in lipid metabolism [34] and can explain some gender differences.

Only males showed an association between *ABCA1*-dependent metabolic parameters (γGT and total cholesterol) and the histological parameters in our study. Lower total cholesterol was associated with severe fibrosis, severe steatosis, and advanced necroinflammation grade. These results are in concordance with other studies because they show that the lower lipid level is an independent predictor of liver fibrosis, necroinflammation, and nonresponse to antiviral treatment [13,32].

The ABCA1 transporter is the principal regulator of cellular cholesterol and phospholipid homeostasis. It moves phospholipids and cholesterol across the cell membrane to combine them with Apolipoprotein AI, which is also synthesized in the liver to form nascent HDL particles [19].

Although with conflicting results, genetic variations in the *ABCA1* gene (such as rs2230808) were described as gender and race-specific. They have also been associated with changes in lipid levels. For example, Allele A of R1578K (Lysine; K) has been consistently associated with low HDL and Apo A levels [21,22].

*ABCA1*_rs2230808 polymorphism was not directly associated with hepatic fibrosis and steatosis. Although, we found an association between this polymorphism and higher values of γGT for those patients that reported more severe liver disease.

A study from 2013 demonstrated severe fibrosis, steatosis, and clinical outcomes (hepatic decompensation, hepatocellular carcinoma, or death) among patients with higher Γgt [35].

Another study showed a significant association between γGT and lipid metabolism, with positive correlations between γGT, LDL, and triglycerides, and a negative correlation between γGT and HDL. Since changes in the *ABCA1* gene can modulate lipid parameters, as referred to before, the association between the studied polymorphism and γGT was expected [36].

Our study confirmed that *ABCA1* polymorphism rs2230808 is gender-specific in its effects, as we obtained different results between males and females regarding the association of this polymorphism with the metabolic and histological profile of patients with CHC. Although our data revealed similar basal fibrosis stages between genders, the gender differences in these genetic and biochemical profiles could explain the different natural histories of HCV disease, like mortality and the presence of concomitant metabolic disorders. Furthermore, HCV is not only hepatotropic, but also lymphotropic. Therefore, HCV’s cytopathic and immunomodulatory mechanisms promote a crosstalk between metabolic disturbances, oxidative stress, and chronic inflammation. These may trigger extra-hepatic manifestations such as cardiovascular disorders, insulin resistance, and hematological malignancies associated with HCV infection [37,38].

Regarding *ABCA1*_rs2230808, females with at least one G allele (GG or GA) showed a higher viral load. In addition, a recent study revealed that pharmacological stimulation of the *ABCA1*-dependent cholesterol efflux pathway disrupts membrane cholesterol homeostasis, leading to the inhibition of virus-cell fusion and, thus, HCV cell entry [39].

The fact that ABCA1 can inhibit the expression of inflammatory factors suggests its relevant role in the interaction between inflammation and reverse cholesterol transport. Its direct and indirect anti-inflammatory mechanisms, including lipid transport, HDL formation, and apoptosis, modulate the immune response and inflammation. On the other hand, some studies demonstrated the role of inflammatory cytokines, proteins, lipids, and the endotoxin-mediated inflammatory process in the expression of ABCA1 [40].

This study analyzed the impacts of *ABCA1* polymorphism on cholesterol metabolism and liver functions in CHC patients. However, this study enrolled only 161 CHC patients, and the number of patients with R1587K substitution in *ABCA1* was only 13.

This fact may be the major limitation of this study, as the impact of this polymorphism on the *ABCA1* gene might be insignificant.

Despite being statistically significant, the relationships found between the studied genetic polymorphism and the metabolic parameters cannot be considered conclusive or irrefutable because the number of patients is insufficient for a minimum statistical power of 0.8. Some validation tests are needed to indicate this study’s accuracy using more study subjects.

However, our results align with some studies already published regarding the role of the ABCA1 transporter in modulating lipid metabolism and its relationship with viral and liver injury markers. They can be a starting point for studies involving a more significant number of CHC patients to obtain sufficient statistical power to validate the results obtained.

## 6. Conclusions

In our study, despite gender differences, the presence of the G allele of *ABCA1*_rs2230808 predicts a more severe liver disease indirectly.

In conclusion, this study confirms the relationship between *ABCA1*_rs2230808 and lipid metabolism, which may also impact the interplay between liver disease and the hematologic and cardiovascular disorders associated with HCV infection, even after eliminating HCV infection.

Despite being statistically significant, the associations found between *ABCA1*_rs2230808 and metabolic changes should be a starting point for more comprehensive studies involving a more substantial number of patients.

## Figures and Tables

**Table 1:** Baseline clinical, biochemical, histological, and genetic parameters of patients.

Parameter	Overall (*n* = 161)	Female (*n* = 60)	Male (*n* = 101)	*p*-Value
Age (years)	46.14 ± 12.46	50.35 ± 13.90	43.62 ± 10.81	0.002 *
BMI (kg/m^2^)	25.44 ± 3.93	25.51 ± 5.39	25.40 ± 2.77	0.151 *
HCV-RNA (UI/mL)	3.25 × 10^5^ [607–4.25 × 10^7^]	2.94 × 10^5^ [7.14 × 10^2^–9.64 × 10^6^]	3.93 × 10^5^ [6.07 × 10^2^–4.25 × 10^7^]	0.103 **
HCV genotype 1 or 4	122 (82.4)	46 (82.1)	76 (82.6)	1.000 ***
HCV genotype 2 or 3	26 (17.6)	10 (17.9)	16 (17.4)	
AP (UI/L)	69.00 [27.00–268.00]	70.00 [27.00–157.00]	68.50 [34.00–268.00]	0.990 **
AST (UI/L)	47.50 [19.00–71.00]	47.00 [19.00–367.00]	48.00 [20.00–243.00]	0.759 **
ALT (UI/L)	71.00 [15.00–709.00]	65.50 [15.00–709.00]	74.50 [20.00–505.0]	0.201 **
γGT (UI/L)	43.00 [10.00–536.00]	30.00 [10.00–536.00]	51.00 [13.00–534.00]	0.001 **
Platelets	2.07 × 10^5^ ± 2.05 × 10^5^	2.21 × 10^5^ ± 7.46 × 10^4^	1.97 × 10^5^ ± 8.36 × 10^4^	0.081 *
Total Cholesterol (mmol/L)	4.15 ± 0.88	4.28 ± 0.86	4.07 ± 0.88	0.167 *
Triglycerides (mmol/L)	0.98 [0.30–3.60]	0.90 [0.50–2.95]	1.32 [0.33–3.60]	0.083 **
HDL (mmol/L)	1.27 [0.15–29.00]	1.45 [0.22–2.60]	1.64 [0.15–29.00]	<0.001 **
LDL (mmol/L)	2.45 ± 0.70	2.53 ± 0.70	2.41 ± 0.70	0.431 *
Fibrosis stage (F1/2)	110 (75.3)	44 (81.5)	66 (71.7)	0.263 ***
Fibrosis stage (F3/4)	36 (24.7)	10 (18.5)	26 (28.3)	
Necroinflammatory activity grade (1–3)	25 (29.4)	11 (40.7)	14 (24.1)	0.191 ***
Necroinflammatory activity grade (4–6)	60 (70.6)	16 (59.3)	44 (75.9)	
Steatosis grade (0)	21 (29.6)	5 (23.8)	16 (32.0)	0.292 ***
Steatosis grade (1–2)	34 (47.9)	13 (61.9)	21 (42.0)	
Steatosis grade (3–4)	16 (22.5)	3 (14.3)	13 (26.0)	
*ABCA1*_rs2230808_GG	92 (57.1)	36 (60.0)	56 (55.4)	
*ABCA1*_rs2230808_GA	56 (34.8)	21 (35.0)	35 (34.7)	0.533 ***
*ABCA1*_rs2230808_AA	13 (8.1)	3 (5.0)	10 (9.9)	

* Independent samples *t*-Test; ** Mann-Whitney Test; *** Chi-squared test; Bold for significant results.

**Table 2:** Association of *ABCA1*_rs2230808 with biochemical and histological parameters.

	Female				Male			
Parameter	*ABCA1*_rs2230808			*p*-Value	*ABCA1*_rs2230808			*p*-Value
	GG	GA	AA		GG	GA	AA	
	Median [Min–Max]/Average ± Standard Deviation (*n*)				Median [Min–Max]/Average ± Standard Deviation (*n*)			
HCV-RNA (UI/mL)	9.6 × 10^5^ [7.1 × 10^2^–8.2 × 10^6^] (31)	10 × 10^5^ [3.2 × 10^3^–9.6 × 10^6^] (18)	0.2 × 10^5^ [2.9 × 10^3^–2.9 × 10^4^] (3)	0.062 **	3.2 × 10^5^ [6.1 × 10^2^–4.3 × 10^7^] (53)	3.6 × 10^5^ [6.1 × 10^2^–1.2 × 10^7^] (30)	5.5 × 10^5^ [8.6 × 10^4^–1.0 × 10^7^] (7)	0.927 **
AP (UI/L)	70.47 ± 29.23 (28)	76.83 ± 16.70 (18)	- (0)	0.166 *	79.81 ± 42.53 (42)	73.72 ± 24.14 (26)	72.80 ± 30.86 (10)	0.742 *
AST (UI/L)	47 [21–193] (34)	45 [19–367] (20)	72 [50–93] (2)	0.621 **	48 [20–199] (54)	48 [27–243] (30)	43 [25–150] (10)	0.882 **
ALT (UI/L)	66 [20–275] (34)	72 [15–709] (20)	88 [53–122] (2)	0.894 **	78 [21–430] (54)	73 [20–505] (30)	64 [36–291] (10)	0.779 **
γGT (UI/L)	31 [10–536] (34)	25 [10–174] (19)	26 [18–34] (2)	0.787 **	55 [15–534] (54)	46 [13–155] (28)	24 [15–126] (10)	0.033 **
Platelets	2.24 × 10^5^ ± 6.67 × 10^4^ (33)	2.20 × 10^5^ ± 7.55 × 10^4^ (21)	1.93 × 10^5^ ± 1.62 × 10^4^ (3)	0.799 *	1.92 × 10^5^ ± 7.32 × 10^4^ (50)	1.98 × 10^5^ ± 7.51 × 10^3^ (31)	2.27 × 10^5^ ± 1.57 × 10^5^ (8)	0.554 *
Total Cholesterol (mmol/L)	4.24 ± 0.94 (31)	4.40 ± 0.74 (18)	3.90 ± 0.86 (2)	0.680 *	3.01 ± 0.81 (52)	4.01 ± 0.85 (27)	4.20 ± 1.44 (7)	**<0.001** *
Triglycerides (mmol/L)	0.94 [0.50–2.80] (31)	0.94 [0.52–2.95] (18)	0.70 [0.66–0.75] (2)	0.387 **	1.10 [0.38–3.60] (51)	0.96 [0.33–3.49] (27)	1.13 [0.52–3.47] (7)	0.501 **
HDL (mmol/L)	1.55 ± 0.42 (21)	1.40 ± 0.43 (13)	- (1)	0.598 *	1.05 [0.15–4.10] (38)	1.05 [0.64–29] (20)	1.06 [0.29–1.35] (5)	0.496 **
LDL (mmol/L)	2.42 ± 0.62 (17)	2.71 ± 0.86 (10)	- (1)	0.590 *	2.40 ± 0.71 (34)	2.39 ± 0.54 (18)	2.49 ± 1.22 (5)	0.963 *
Fibrosis stage (F1/2)	27 (87.1)	16 (80.0)	1 (33.3)	0.071 ***	34 (66.7)	23 (74.2)	9 (90)	0.304 ***
Fibrosis stage (F3/4)	4 (12.9)	4(20.0)	2 (66.7)		17 (33.3)	8 (25.8)	1 (10.0)	
Necroinflammatory activity grade (1–3)	7 (63.6)	4 (36.4)	0 (0.0)	0.483 ***	7 (50.0)	3 (21.4)	4 (28.6)	0.285 ***
Necroinflammatory activity grade (4–6)	7 (43.8)	8 (50.0)	1 (6.3)		25 (56.8)	14 (31.8)	5 (11.4)	
Steatosis grade (1–2)	6 (46.2)	6 (46.2)	1 (7.7)	0.768 ***	12 (57.1)	6 (28.6)	3 (14.3)	0.749 ***
Steatosis grade (3–4)	2 (66.7)	1 (33.3)	0 (0.0)		9 (69.2)	3 (23,1)	1 (7.7)	

* One way ANOVA Test; ** Kruskal-Wallis Test; *** Chi-squared test; Bold for significant results.

**Table 3:** Association of *ABCA1*_rs2230808 with biochemical and histological parameters; dominant model (GG or GA vs. AA).

	Female			Male		
Parameter	*ABCA1*_rs2230808			*ABCA1*_rs2230808		
	GG or GA	AA	*p*-Value	GG or GA	AA	*p*-Value
	Median [Min–Max]/Average ± Standard Deviation (*n*)			Median [Min–Max]/Average ± Standard Deviation (*n*)		
HCV-RNA (UI/mL)	9.8 × 10^5^ [7.1 × 10^2^–9.6 × 10^6^] (49)	0.2 × 10^5^ [2.9 × 10^3^–2.9 × 10^4^] (3)	**0.013** **	5.5 × 10^5^ [8.6 × 10^4^–4.2 × 10^7^] (83)	5.5 × 10^5^ [8.6 × 10^4^–1.0 × 10^7^] (7)	0.701 **
AP (UI/L)	67.71 ± 25.06 (28)	- (0)	-	77.50 ± 30.50 (68)	72.80 ± 30.86 (10)	0.700 *
AST (UI/L)	47 [19–367] (54)	72 [50–93] (2)	0.421 **	48 [20–243] (84)	43 [25–150] (10)	0.619 **
ALT (UI/L)	66 [15–709] (54)	88 [53–122] (2)	0.687 **	77 [20–505] (84)	64 [36–291] (10)	0.485 **
γGT (UI/L)	30 [10–536] (53)	26 [18–34] (2)	0.594 **	52 [13–534] (82)	24 [15–126] (10)	**0.017** **
Platelets	2.23 × 10^5^ ± 6.96 × 10^4^ (54)	1.93 × 10^5^ ± 1.62 × 10^4^ (3)	0.786 *	1.94 × 10^5^ ± 7.35 × 10^4^ (81)	2.27 × 10^5^ ± 1.57 × 10^5^ (8)	0.585 *
Total Cholesterol (mmol/L)	4.30 ± 0.86 (49)	3.90 ± 0.86 (2)	0.520 *	3.50 ± 0.81 (79)	4.20 ± 1.44 (7)	**0.044** *
Triglycerides (mmol/L)	0.94 [0.50–2.95] (49)	0.70 [0.66–0.75] (2)	0.265 **	1.06 [0.33–3.60] (78)	1.13 [0.52–3.47] (7)	0.817 **
HDL (mmol/L)	1.50 ± 0.42 (27)	1.55 (1)	^-^	1.06 [0.15–29] (5)	1.06 [0.29–1.35] (5)	0.859 **
LDL (mmol/L)	2.53 ± 0.72 (27)	- (1)	0.881 *	2.40 ± 0.65 (52)	2.49 ± 1.22 (5)	0.877 *
Fibrosis stage (F1/2)	43 (84.3)	1 (33.3)	0.085 ***	57 (69.5)	9 (90)	0.272 ***
Fibrosis stage (F3/4)	8 (15.7)	2 (66.7)		25 (30.5)	1 (10.0)	
Necroinflammatory activity grade (1–3)	11 (42.3)	0 (0.0)	1.000 ***	10 (20.4)	4 (28.6)	0.198 ***
Necroinflammatory activity grade (4–6)	15 (57.7)	1 (6.3)		39 (79.6)	5 (11.4)	
Steatosis grade (1–2)	12 (80.0)	1 (7.7)	1.000 ***	18 (60.0)	3 (14.3)	1.000 ***
Steatosis grade (3–4)	3 (20.0)	0 (0.0)		12 (40.0)	1 (7.7)	

* Independent samples *t*-Test; ** Mann-Whitney Test; *** Chi-squared test; Bold for significant results.

**Table 4:** Association of biochemical and histological parameters.

**(a)**									
**Parameter**	**Female**								
	**Fibrosis Stage** **(F1/2)**	**Fibrosis Stage** **(F3/4)**	***p*** **-Value**	**Steatosis Grade** **(1–2)**	**Steatosis Grade** **(3–4)**	***p*** **-Value**	**Necroinflammatory** **Activity Grade** **(1–3)**	**Necroinflammatory** **Activity Grade** **(4–6)**	***p*** **-Value**
	**Median [Min–Max]/Average ± Standard Deviation** **(*n*)**			**Median [Min–Max]/Average ± Standard Deviation** **(*n*)**			**Median [Min–Max]/Average ± Standard Deviation** **(*n*)**		
HCV-RNA (UI/mL)	3.01 × 10^5^ [7.12 × 10^2^–9.63 × 10^6^] (39)	2.2 × 10^5^ [2.90 × 10^3^–1.71 × 10^6^] (9)	0.741 **	3.61 × 10^5^ [1.55 × 10^4^–5.16 × 10^6^] (11)	5.86 × 10^5^ [1.51 × 10^4^–1.31 × 10^6^] (3)	0.769 **	2.13 × 10^5^ [7.14 × 10^2^–9.64 × 10^6^] (9)	4.05 × 10^5^ [1.48 × 10^4^–3.21 × 10^6^] (16)	0.396 **
AP (UI/L)	72.23 ± 25.79 (39)	85.57 ± 22.54 (7)	0.148 *	81.40 ± 32.30 (10)	85.33 ± 49.14 (3)	0.871 *	67.18 ± 18.33 (11)	79.00 ± 36.85 (11)	0.352 *
AST (UI/L)	41 [19–164] (43)	82 [41–367] (10)	**0.001** **	47 [30–144] (13)	90 [34–133] (3)	0.611 **	44 [26–146] (11)	67 [33–367] (16)	0.098 **
ALT (UI/L)	56 [15–295] (43)	111 [53–709] (10)	**0.005** **	83 [37–182] (13)	114 [69–190] (3)	0.364 **	56 [19–269] (11)	103 [37–507] (16)	**0.026** **
γGT (UI/L)	25 [10–536] (42)	33 [17–118] (10)	0.341 **	34 [11–536] (13)	62 [53–69] (3)	0.082 **	21 [10–153] (11)	37 [17–536] (15)	**0.038** **
Platelets	2.34 × 105 ± 7.18 × 104 (42)	1.75 × 105 ± 7.53 × 104 (10)	**0.042** *	2.00 × 105 ± 6.61 × 104 (12)	1.42 × 105 ± 3.49 × 104 (3)	0.174 *	2.23 × 105 ± 6.47 × 104 (10)	2.04 × 105 ± 37.36 × 104 (15)	0.386 *
Total Cholesterol (mmol/L)	4.32 ± 0.85 (38)	4.02 ± 0.83 (10)	0.312 *	4.34 ± 1.08 (12)	4.94 ± 1.58 (3)	0.436 *	4.53 ± 10.70 (10)	4.34 ± 1.10 (15)	0.638 *
Triglycerides (mmol/L)	0.89 [0.56–2.95] (38)	0.95 [0.59–1.54] (10)	0.800 **	0.96 [0.52–5.95] (12)	1.51 [1.24–2.46] (3)	0.101 **	0.78 [0.52–2.80] (10)	0.99 [0.59–2.95] (15)	0.085 **
HDL (mmol/L)	1.58 ± 0.37 (23)	1.32 ± 0.53 (9)	0.125 *	1.52 ± 0.54 (9)	2.23 (1)	0.244 *	1.50 ± 0.41 (5)	1.52 ± 0.56 (9)	0.956 *
LDL (mmol/L)	2.51 ± 0.63 (18)	2.60 ± 0.77 (8)	0.745 *	2.81 ± 0.96 (5)	- (0)	-	2.23 ± 0.64 (4)	2.86 ± 0.92 (5)	0.288 *
**(b)**									
**Parameter**	**Male**								
	**Fibrosis Stage** **(F1/2)**	**Fibrosis Stage** **(F3/4)**	***p*-Value**	**Steatosis Grade** **(1–2)**	**Steatosis Grade** **(3–4)**	***p*-Value**	**Necroinflammatory** **Activity Grade** **(1–3)**	**Necroinflammatory** **Activity Grade** **(4–6)**	***p*** **-Value**
	**Median [Min–Max]/Average ± Standard Deviation** **(*n*)**			**Median [Min–Max]/Average ± Standard Deviation** **(*n*)**			**Median [Min–Max]/Average ± Standard Deviation** **(*n*)**		
HCV-RNA (UI/mL)	2.91 × 10^5^ [6.07 × 10^2^–1.00 × 10^7^] (60)	6.50 × 10^5^ [1.41 × 10^5^–1.18 × 10^7^] (23)	**0.025** **	1.74 × 10^5^ [6.07 × 10^2^–1.08 × 10^7^] (19)	7.31 × 10^5^ [1.41 × 10^5^–1.00 × 10^7^] (12)	**0.011** **	4.49 × 10^5^ [5.73*10^3^–2.89 × 10^6^] (13)	2.93 × 10^5^ [6.07 × 10^2^–1.08 × 10^7^] (39)	0.254 **
AP (UI/L)	73.28 ± 30.31 (53)	86.74 ± 49.83 (19)	0.171*	70.15 ± 25.86 (20)	104.36 ± 60.53 (11)	**0.035** *	64.43 ± 12.85 (14)	79.92 ± 36.67 (37)	**0.030** *
AST (UI/L)	44 [20–243] (64)	83 [27–199] (23)	**<0.001** **	40 [20–150] (21)	54 [33–199] (11)	**0.012** **	37 [26–58] (14)	44 [24–243] (42)	0.173 **
ALT (UI/L)	69 [20–505] (64)	119 [44–327] (23)	**<0.001** **	74 [32–211] (21)	119 [33–430] (11)	**0.037** **	68 [32–135] (14)	76 [20–505] (42)	0.233 **
γGT (UI/L)	49 [13–265] (62)	118 [22–534] (23)	**0.002** **	44 [13–150] (21)	118 [22–167] (11)	**0.010** **	46 [22–141] (14)	54 [13–265] (40)	0.921 **
Platelets	2.08 × 10^5^ ± 8.35 × 10^4^ (61)	1.55 × 10^5^ ± 8.10 × 10^4^ (10)	**0.014** *	1.92 × 10^5^ ± 6.29 × 10^4^ (19)	1.97 × 10^5^ ± 5.92 × 10^4^ (10)	0.852 *	2.29 × 10^5^ ± 1.25 × 10^5^ (12)	2.11 × 10^5^ ± 6.42 × 10^4^ (10)	0.517 *
Total Cholesterol (mmol/L)	4.22 ± 0.85 (58)	3.72 ± 0.93 (22)	**0.024** *	4.22 ± 0.86 (20)	3.44 ± 0.68 (10)	**0.019** *	4.52 ± 1.10 (14)	4.09 ± 0.86 (37)	0.148 *
Triglycerides (mmol/L)	1.04 [0.38–3.60] (57)	1.12 [0.33–3.20] (22)	0.626 **	1.13 [0.56–3.19] (20)	1.30 [0.63–3.47] (10)	0.559 *	1.13 [0.38–2.38] (14)	1.03 [0.43–3.60] (37)	0.966 **
HDL (mmol/L)	1.85 ± 0.26 (43)	1.19 ± 0.85 (17)	0.526 *	1.06 ± 0.17 (12)	1.58 ± 1.43 (5)	0.463 *	1.09 ± 0.45 (8)	1.18 ± 0.57 (25)	0.680 *
LDL (mmol/L)	2.57 ± 0.70 (38)	2.04 ± 0.62 (16)	**0.011** *	2.51 ± 0.90 (11)	2.08 ± 0.60 (3)	0.373 *	2.78 ± 0.73 (7)	2.43 ± 0.82 (21)	0.324 *

* Independent samples *t*-Test; ** Mann-Whitney Test; Bold for significant results.

## Data Availability

The datasets used and analyzed during the current study are available from the corresponding author upon reasonable request.
